# Comparison of Prognosis Between Microscopically Positive and Negative Surgical Margins for Primary Gastrointestinal Stromal Tumors: A Systematic Review and Meta-Analysis

**DOI:** 10.3389/fonc.2022.679115

**Published:** 2022-04-19

**Authors:** Zhen Liu, Yichunzi Zhang, Han Yin, Xiuzhu Geng, Sishang Li, Jinrong Zhao, Ziyang Zeng, Xin Ye, Jianchun Yu, Fan Feng, Weiming Kang

**Affiliations:** ^1^ Department of General Surgery, Peking Union Medical College Hospital, Chinese Academy of Medical Sciences & Peking Union Medical College, Beijing, China; ^2^ National Health Commission (NHC) Key Laboratory of Systems Biology of Pathogens and Christophe Mérieux Laboratory, Institute of Pathogen Biology, Chinese Academy of Medical Sciences & Peking Union Medical College, Beijing, China; ^3^ Ministry of Health (MOH) Key Laboratory of Systems Biology of Pathogens, Institute of Pathogen Biology, and Center for Tuberculosis Research, Chinese Academy of Medical Sciences & Peking Union Medical College, Beijing, China; ^4^ Department of Hematology, Peking Union Medical College Hospital, Chinese Academy of Medical Sciences & Peking Union Medical College, Beijing, China; ^5^ Division of Digestive Surgery, Xijing Hospital of Digestive Diseases, The Air Force Medical University, Xi’an, China

**Keywords:** gastrointestinal stromal tumor, R1 margin, Imatinib, prognosis, meta-analysis

## Abstract

**Background:**

This meta-analysis aimed to determine the prognostic impact of microscopically positive margins (R1) on primary gastrointestinal stromal tumors.

**Methods:**

A literature search was performed using PubMed, Embase, Web of Science, and Cochrane Library for studies up to 23 November 2020. The pooled disease-free survival (DFS) and overall survival (OS) between R1 and negative margins (R0) were estimated using a random-effects model.

**Results:**

Twenty studies with 6,465 patients were included. Compared with R0 resection, R1 was associated with poor DFS in patients who did not receive adjuvant Imatinib (HR: 1.62, 95% CI: 1.26–2.09; P = 0.48, I^2^ = 0%; reference: R0). This negative impact of R1 disappeared with the use of adjuvant Imatinib (HR: 1.23, 95% CI: 0.95–1.60; P = 0.38, I2 = 6%; reference: R0). R1 was related to poor DFS in gastric GISTs (HR: 2.15, 95% CI: 1.15–5.02, I^2^ = 0%; reference: R0), which was attenuated in the subgroup of adjuvant Imatinib (HR: 2.24, 95% CI: 0.32–15.60; P = 0.84, I^2^ = 0%; reference: R0). Rectal GIST with R1 margin who even received adjuvant Imatinib still had poor DFS (HR: 3.79, 95% CI: 1.27–11.31; P = 0.54, I^2^ = 0%; reference: R0). Patients who underwent R1 resection had similar OS compared with those underwent R0 resection regardless of the use of adjuvant Imatinib.

**Conclusion:**

R1 was associated with poor DFS for primary GISTs, which was attenuated by adjuvant therapy with Imatinib. Similar result was observed in the gastric GISTs subgroup. Rectal GIST patients with R1 resection had poor DFS even when they received adjuvant Imatinib. The R1 margin did not influence the OS of GISTs.

## Introduction

Gastrointestinal stromal tumors (GISTs) are one of the most common mesenchymal tumors, accounting for 80% of tumors that arise from the gastrointestinal tract (1). The rare invasion to lymph nodes or adjacent organs that occurs with these tumors makes it possible to perform a local resection as a radical curative treatment, which requires a negative resection margin (R0) and avoidance of tumor rupture to achieve a satisfactory oncological outcome (2, 3). However, incomplete resection might occur in cases with tumors located in unfavorable anatomical sites, which results in microscopically or grossly positive resection margins (R1). With the advent of minimally invasive procedures, such as laparoscopy and endoscopy, whether the status of resection margin impacts oncological outcomes of GISTs remains a core concern for surgeons.

Several studies (4–7) have evaluated the prognostic value of R1 margin for GIST, through which controversial results were drawn out partially because of the retrospective nature or the relatively small sample size of these studies. The only previous meta-analysis (8) revealed that adjuvant Imatinib could attenuate the negative influence of R1 resection on disease-free survival (DFS) of GISTs. However, a recent *post hoc* study based on the EORTC 62024 randomized trial suggested that tumor rupture rather than R1 margin significantly influenced the overall survival (OS) of GIST regardless of the acceptance of adjuvant Imatinib (9). To date, high-quality evidence focusing on this issue is still lacking, which is why a decisive conclusion remains unclear. Therefore, the current meta-analysis aimed to review the current literature and provide a comprehensive perspective on the influence of the R1 margin on the prognosis of GIST.

## Materials and Methods

### Search Strategy

A systematic search of literature using keywords such as “gastrointestinal stromal tumor,” “GIST,” “margin,” and “R1” was carried out by two investigators (ZL and YZ) through PubMed, Embase, Web of Science, and Cochrane Library to identify studies that reported the relationship between the status of surgical margins and prognosis of gastrointestinal stromal tumor. The search included studies up to 23 November 2020. Attempts have been made to obtain additional eligible studies by searching the references of relevant studies. This study adhered to the Preferred Reporting Items for Systematic Reviews and Meta-analyses (PRISMA) guidelines (10).

### Selection Criteria

Eligible studies were identified by two investigators (ZL and HY) according to the following criteria: (1) Participants (P): The patients were diagnosed pathologically and immunohistochemically as primary GISTs without metastasis or other cancers; (2) Interventions (I) and comparisons (C): All the patients underwent surgery and outcomes between R1 and R0 resection margin were compared; (4) Outcomes (O): DFS and/or OS were available or able to be calculated by sufficient data in the studies. When duplicate studies based on similar populations were identified, only the newest or largest study was included. Any discrepancies were resolved by discussion with a third investigator (XG).

### Data Extraction

The name of the first author, year of publication, country, sample size, tumor site, recurrence events, adjuvant therapy, follow-up, DFS, disease-specific survival, and OS were extracted independently by two investigators (SL and JZ). If the hazard ratio (HR) and 95% confidence interval (CI) were not provided in the studies, we calculated these data from available data or from the Kaplan–Meier survival curves using the methods reported by Tierney et al. (11). A third observer (ZZ) engaged in discussions to resolve any controversial issues.

### Quality Assessment

Two authors (ZL and ZZ) independently assessed the quality of all included studies using the Newcastle–Ottawa Quality Assessment Scale (NOS) with the highest score of nine (12), and any discrepancies in the scores were resolved by discussion with a third reviewer (YZ).

### Statistical Analysis

The pooled survival data were measured using the HR and 95% CI. Some HRs and 95% CIs were extracted from Kaplan–Meier curves using Engauge Digitizer (version 4.1). Statistical heterogeneity was evaluated using the chi-square test and I^2^ statistics. Subgroup analysis was conducted to identify the source of heterogeneity. The random-effects model was used by default because of the nature of the included studies. The estimated results of the fixed-effects model are also provided for reference. Sensitivity analysis was performed to validate the stability of the model by sequentially omitting each study. Potential publication bias was assessed using the Begg’s and Egger’s tests. Statistical analyses were performed using R software 3.6.1 (R Project for Statistical Computing) with the meta package (4.13-0) (13). A two-sided P <0.05 was considered significant. The GRADE profiler software (version 3.6) was used to estimate the level of evidence (14).

## Results

### Eligible Studies and Characteristics

**Figure 1:**
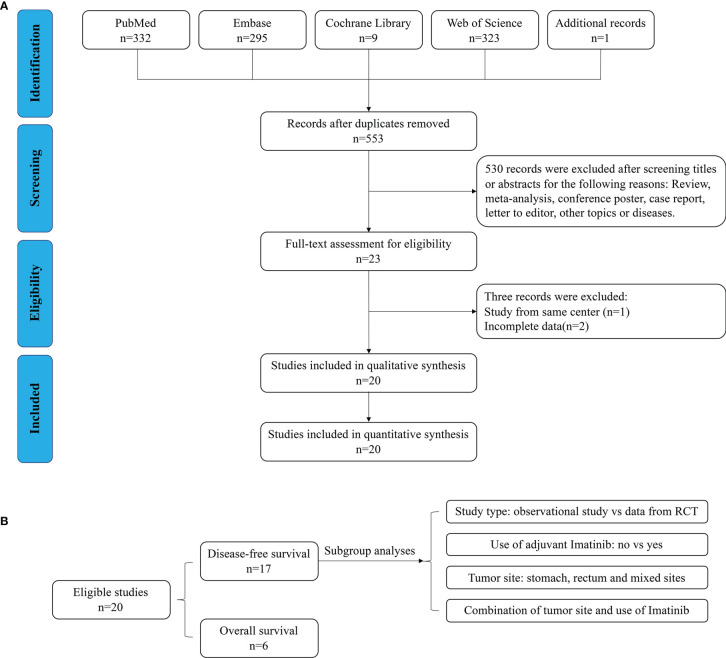
Flow chart of **(A)** search strategy and **(B)** study design.

As shown in **Figure 1A**, 960 relevant publications were identified in the literature search. After screening and assessment, a total of 20 eligible studies (6, 7, 9, 12, 15–30) with 6,465 patients were included in this meta-analysis (**Table 1**). In their studies, McCarter and Cavnar analyzed two sub-series of patients with GIST with or without adjuvant Imatinib. Therefore, the final analysis involved 22 series from 20 studies. There were 5,662 patients who underwent R0 resection, and 803 patients who underwent R1 resection. A total of 915 patients experienced recurrence after R0 resection, while 159 patients who underwent R1 resection experienced recurrence. Adjuvant Imatinib was prescribed to patients in 13 studies. The NOS scores of the studies ranged from seven to eight, indicating their relatively high quality of methodology. The DFS and OS of GIST between R1 and R0 resection were compared, and the subgroup analyses, according to study type, use of adjuvant Imatinib, and tumor site (**Figure 1B**).

**Table 1:** Summarization of the included studies.

Study	Country	Type	Site	Sample size			IM	Follow-up (median)	Disease-freee survival	Overall survival	NOS
				Total	R0/Recur	R1/Recur					
DeMatteo et al., (6)	USA	Observ	Mix	80	65/NA	15/NA	No	24 (1–175) mo	NA	2.69 (0.67, 10.89)^***^	7
Pierie et al., (15)	USA	Observ	Mix	39	35/13	4/3	No	38 (1–159) mo	1.44 (0.29, 7.24)	NA	7
Rutkowski et al., (12)	Poland	Observ	Mix	328	253/102	75/46	No	31 (4–292) mo	1.62 (1.12, 2.35)^**^	NA	7
Gouveia et al., (16)	Portugal	Observ	Mix	96	78/7	18/5	No	42 (1–206) mo	3.03 (0.96, 9.56)^**^	1.54 (0.34, 7.08)^***^	7
Nikfarjam et al., (17)	USA	Observ	Mix	40	35/15	5/1	Yes	24 (1–74) mo	0.81 (0.18, 3.55)	NA	7
Catena et al., (18)	Italy	Observ	Stomach	151	132/NA	19/NA	No	101 (11–132) mo	2.4 (1.1, 4.3)^**^	NA	7
Huang et al., (19)	China	Observ	Stomach	85	82/24	3/1	Yes	41 (3–100) mo	2.04 (0.24, 17.03)	NA	7
Kim et al., (20)	Korea	Observ	Stomach	136	122/5	14/0	No	29 (3–106) mo	0.3 (0.02, 5.45)	NA	7
McCarter_Placebo et al., (21)^*^	USA	RCT	Mix	353	330/90	23/9	No	49 mo	1.5 (0.76, 2.99)^**^	NA	8
McCarter_Imatinib et al., (21)^*^	USA	RCT	Mix	464	415/114	49/17	Yes	49 mo	1.1 (0.66, 1.83)^**^	NA	8
Jakob et al., (22)	Germany	Observ	Rectum	16	14/NA	2/NA	Yes	41 (3–110) mo	1.27 (0.03, 49.2)	NA	7
Ahlen et al., (23)	Sweden	Observ	Mix	79	61/16	18/15	No	76 (10–179) mo	2.58 (0.75, 8.87)	3.94 (0.24, 64.1)^***^	7
Hølmebakk et al., (7)	Norway	Observ	Mix	410	363/53	47/17	Yes	45 (0–175) mo	1.08 (0.6, 1.95)^**^	NA	7
Cavnar_Neo-IM et al., (24)	USA	Observ	Mix	76	64/NA	12/NA	Yes	3.05 (0.01–14.3) y	NA	0.36 (0.05, 2.8)	7
Gronchi et al., (9)	Multi-centers	RCT	Mix	808	743/225	65/29	Yes	9.1(IQR, 8–10) y	1.35 (0.91, 1.99)^**^	1.05 (0.54, 2.01)	7
Pantuso et al., (25)	Italy	Observ	Mix	74	54/12	20/2	Yes	53 (4–117) mo	0.35 (0.11, 1.14)	NA	7
Şenol et al., (26)	Turkey	Observ	Mix	60	51/8	9/3	Yes	47.12 ± 33.52 mo	2.63 (0.31, 22.26)	NA	7
Shannon et al., (27)	USA	Observ	Mix	2,084	2027/231	57/10	Yes	NA	NA	1.26 (0.66, 2.4)	8
Shu et al., (28)	China	Observ	Rectum	71	56/NA	15/NA	Yes	84 mo	4.21 (1.34, 13.21)^**^	NA	7
Zhu et al., (29)	China	Observ	Stomach	371	85/0	286/1	Yes	34.2 ± 20.2 mo	3.52 (0.03, 373.1)	NA	8
Cavnar_pre-IM et al., (30)^*^	USA	Observ	Mix	137	121/NA	16/NA	No	4.6 (0–29) y	1.01 (0.58, 2.07)^**^	NA	7
Cavnar_IM et al., (30)^*^	USA	Observ	Mix	507	476/NA	31/NA	Yes	4.6 (0–29) y	1.29 (0.63, 2.65)^**^	NA	7

Recur, Recurrence; Observ, Observational study including retrospective or prospective study; RCT, Data from RCTs; IM, Adjuvant Imatinib therapy; y, year; mo, month; NA, not available. Mix, Studies that analyzed more than one tumor site.

^*^McCarter and Cavnar each in their studies analyzed two sub-datasets of GIST patients either received Imatinib or not.

^**^Data of survival extracted directly from the original articles.

^***^Disease-specific survival which were further analyzed in combination with overall survival.

### Disease-Free Survival

**Figure 2:**
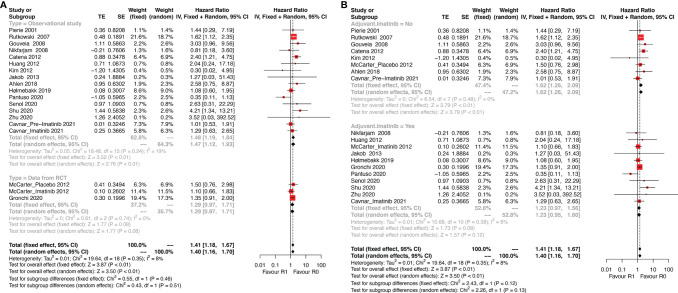
Forest plots illustrating disease-free survival between R1 and R0 margins. Subgroup analysis according to **(A)** study type and **(B)** use of Imatinib.

As shown in **Figure 2**, DFS data between R1 and R0 resection were available in 17 studies (19 series). R1 resection was associated with poor DFS compared with R0 resection (HR: 1.40, 95% CI: 1.16–1.70; reference: R0), which was consistent with the estimated results of the fixed-effects model (HR: 1.41, 95% CI: 1.18–1.67; reference: R0), indicating a lack of heterogeneity among studies (P = 0.35, I^2^ = 8%). Sensitivity analysis was performed by omitting each study sequentially, and the estimated results did not differ significantly, indicating the stability of the model (**Supplementary Figure 1A**).

Two of the 17 studies (three series) analyzed data from randomized controlled trials (RCT) and the remaining 15 were observational studies. Thus, subgroup analysis according to the type of study (observational study vs. RCT, **Figure 2A**) was performed. The results showed that R1 resection was related to poor DFS in the subgroup of observational studies (HR: 1.47, 95% CI: 1.12–1.93; I^2^ = 19%; reference: R0) but not in subgroup of RCT (HR: 1.29, 95% CI: 0.97–1.93; I^2^ = 0%; reference: R0). However, patients of two series of the three in the subgroup analyzing data from RCTs received adjuvant Imatinib.

Thus, another subgroup analysis was performed according to the use of adjuvant Imatinib (**Figure 2B**). R1 resection was correlated with poor DFS compared with R0 resection (HR: 1.62, 95% CI: 1.26–2.09; P = 0.48, I^2^ = 0%; reference: R0) in the subgroup without adjuvant Imatinib, while the status of resection margin had no significant impact on DFS in the adjuvant Imatinib subgroup (HR: 1.23, 95% CI: 0.95–1.60; P = 0.38, I^2^ = 6%; reference: R0).

**Figure 3:**
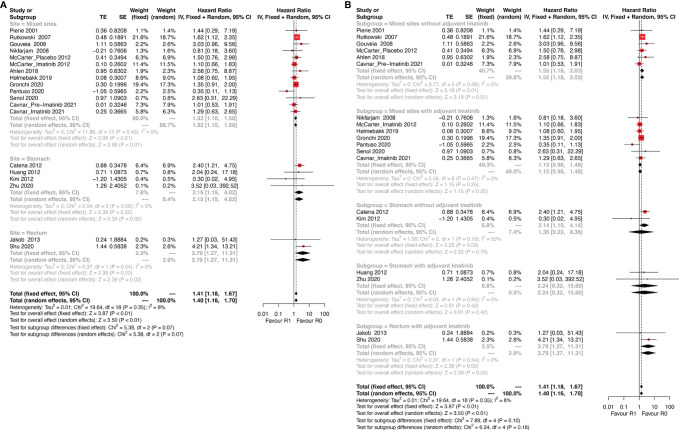
Forest plots illustrating disease-free survival between R1 and R0 margins. Subgroup analysis according to **(A)** tumor site and **(B)** combination of tumor site and use of adjuvant Imatinib.

Tumor site is another key prognostic factor for GISTs. The eligible studies were categorized into three subgroups: stomach, rectum, and mixed sites. The mixed sites included studies that analyzed more than one tumor site. The results of this subgroup analysis (**Figure 3A**) showed that R1 was associated with poor DFS in all three subgroups (stomach: HR: 2.15, 95% CI: 1.15–5.02, I^2^ = 0%; reference: R0; rectum: HR: 3.79, 95% CI: 1.27–11.31; I^2^ = 0%; reference: R0; mixed sites: HR: 1.32, 95% CI: 1.10–1.58; I^2^ = 0%; reference: R0).

The results differed when tumor site and Imatinib use were both taken into consideration (**Figure 3B**). For gastric GIST patients, margin status had no significant influence on DFS regardless of the use of adjuvant Imatinib (without Imatinib: HR: 1.35, 95% CI: 0.22–8.36; P = 0.16, I^2^ = 50%; reference: R0; with Imatinib: HR: 2.24, 95% CI: 0.32–15.60; P = 0.84, I^2^ = 0%; reference: R0). However, a relatively high heterogeneity was observed in the gastric subgroup without adjuvant Imatinib (P = 0.16, I^2^ = 50%), which made the result of this subgroup less reliable. Notably, rectal GIST patients with R1 resection had poor DFS even when they received adjuvant Imatinib (HR: 3.79, 95% CI: 1.27–11.31; P = 0.54, I^2^ = 0%; reference: R0). In the mixed sites group, R1 resection was correlated with poor DFS compared with R0 resection (HR: 1.55, 95% CI: 1.18–2.03; P = 0.58, I^2^ = 0%; reference: R0) for patients without adjuvant Imatinib, while the status of resection margin did not impact DFS for patients receiving adjuvant Imatinib (HR: 1.15, 95% CI: 0.90–1.48; P = 0.47, I^2^ = 0%; reference: R0).

### Overall Survival

**Figure 4:**
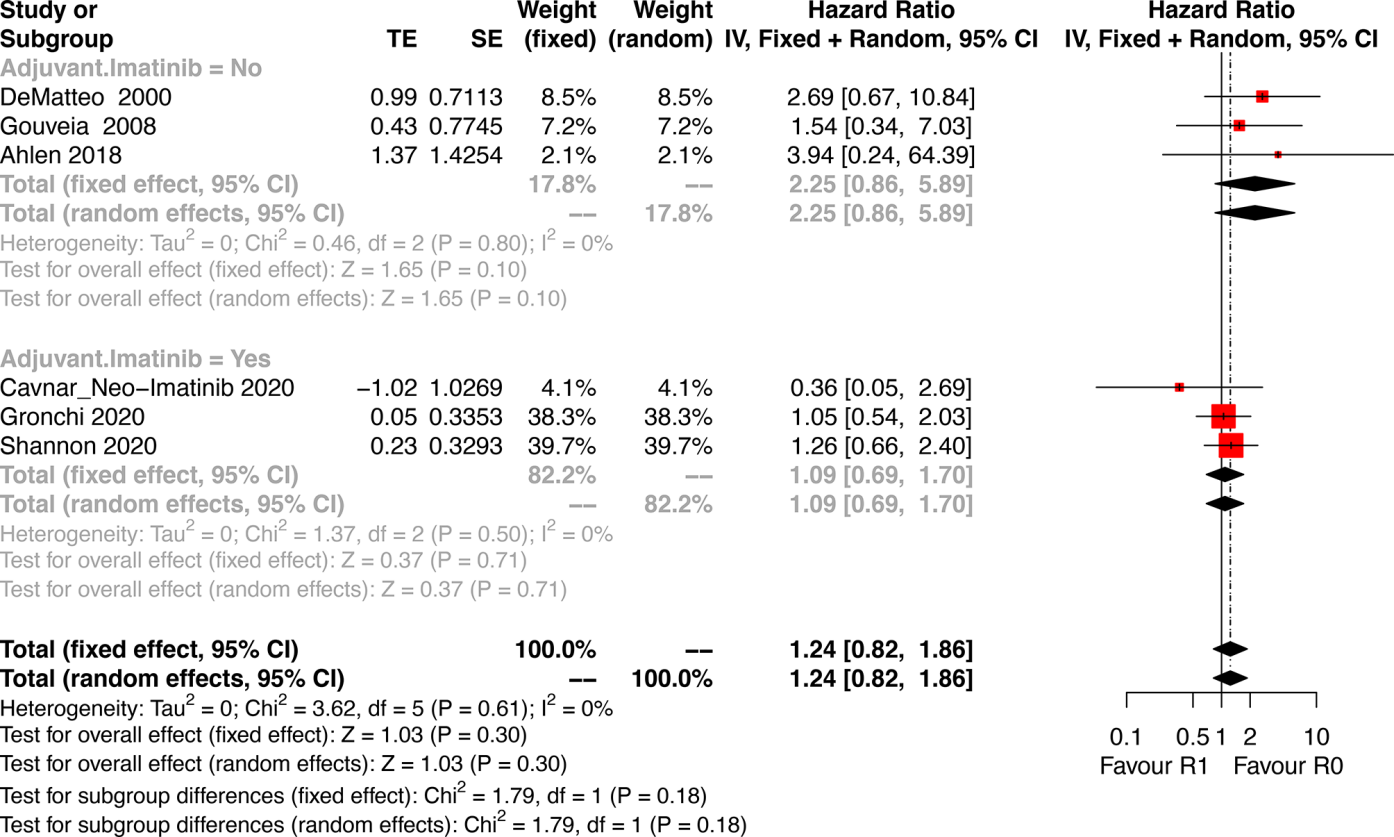
Forest plots illustrating overall survival between R1 and R0 margins.

Six studies that analyzed the OS were included. Patients who underwent R1 resection had similar OS compared with R0 resection (HR: 1.24, 95% CI: 0.82–1.86; P = 0.61, I^2^ = 0%), regardless of whether they received adjuvant Imatinib (HR: 1.09, 95% CI: 0.69–1.70; P = 0.50, I^2^ = 0%) or not (HR: 2.25, 95% CI: 0.86–5.89; P = 0.80, I^2^ = 0%) (**Figure 4**). The estimated results did not significantly differ after omitting each study sequentially, indicating the stability of the model (**Supplementary Figure 1B**).

### Publication Bias and GRADE Quality of Evidence

**Figure 5:**
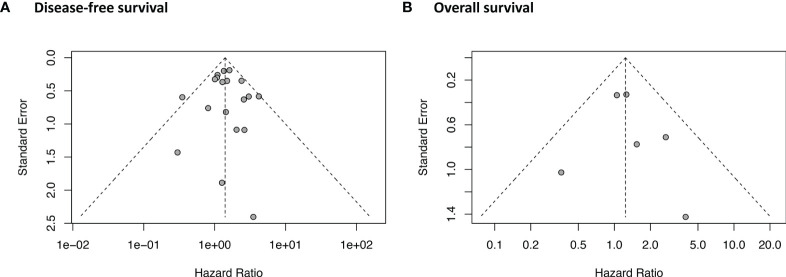
Publication bias of **(A)** disease-free survival and **(B)** overall survival.

As shown in **Figure 5**, the funnel plot and Egger’s test (P = 0.84) indicated that no potential publication bias was detected in the DFS data. No asymmetry was observed in the funnel plot of OS. Egger’s test was not performed for OS because of the relatively small number of studies (n = 6). The GRADE evidence profiles of the two indicators (DFS and OS) are presented in **Table 2**.

**Table 2:** GRADE profile evidence.

Indicators	Quality assessment							№. of patients		Effect	Quality	Importance
	№. of studies	Study design	Risk of bias	Inconsistency	Indirectness	Imprecision	Other considerations	R1	R0	Relative (95% CI)		
DFS	17	observational studies^*^	not serious	not serious	not serious	not serious	Tumor site might influence the effect of R1	719	3,506	HR 1.40 (1.16–1.70)	⨁⨁⨁⊝ moderate	critical
OS	6	observational studies^**^	not serious	not serious	not serious	not serious	none	185	3,038	HR 1.24 (0.82–1.86)	⨁⨁⊝⊝ low	important

*Including two observational studies that analyzed data from two RCTs. **Including one observational study that analyzed data from an RCT.

## Discussion

The present study found that R1 resection was associated with poor DFS for primary GISTs. Subgroup analysis was performed according to study type, use of adjuvant Imatinib, and tumor site. DFS did not worsen for patients who underwent R1 resection in the subgroup of RCT. However, patients of two of the three series in the RCT subgroup received adjuvant Imatinib. To illustrate this point, in the subgroup analysis of the use of adjuvant Imatinib, the negative influence of R1 resection on DFS was attenuated by adjuvant Imatinib. Similar effect of adjuvant Imatinib in DFS was observed in the subgroup of gastric GISTs. Rectal GIST patients who underwent R1 resection had poor DFS even when they received adjuvant Imatinib. Patients who underwent R1 resection had similar OS compared with those underwent R0 resection regardless of the use of adjuvant Imatinib.

Although surgical margin was removed from the 2014 edition of the European Society for Medical Oncology (ESMO) guidelines (31) as a prognostic factor for GIST, debates around this point have not diminished. Consistent with this, a recent study evaluating 371 cases of GIST that were all endoscopically resected and the majority of which were very low or low risk, showed that the R1 margin was not associated with a higher rate of recurrence of GIST. The only previous meta-analysis (8) focusing on resection margins found that the difference in DFS between R1 and R0 margins disappeared in a subgroup of studies in which parts of patients received adjuvant Imatinib, which is recommended for moderate or high-risk patients according to guidelines. The current meta-analysis also found that R1 resection was associated with poor DFS of GISTs, but this negative effect disappeared with use of adjuvant Imatinib. That is to say, in the presence of adjuvant Imatinib, R1 did not negatively impact the DFS of GISTs.

However, the Imatinib in these studies was not specifically given to those who had R1 margins, and the mechanism by which Imatinib attenuated the negative survival impact of R1 requires further exploration. Interestingly, Shannon et al. (27) in their study found that the R1 resection margin was correlated with larger tumor size, which means more aggressive tumor biology that leads to poor prognosis. These results raise the question of whether the prognostic difference is actually caused by the difference in risk factors collinear with the R1 margin rather than the margin status itself. To confirm this point of view, Gronchi et al. (9) analyzed 908 GIST patients from a randomized trial and compared survival between R1 and R0 margins stratified by treatment arm (with or without adjuvant Imatinib). The results showed that when tumor rupture was excluded, the R1 margin was not related to worse relapse-free survival and OS in either arm. The current estimated effect of the R1 margin on the OS of GIST was consistent with this result. However, it could not be simply concluded that margin status did not need to be considered in the decision-making for postoperative treatment of GIST.

Further subgroup analysis of this meta-analysis according to tumor site and use of adjuvant Imatinib showed that gastric GISTs with R1 margin had poor DFS which was attenuated in the subgroup of adjuvant Imatinib. Notably, R1 margin was associated with poor DFS of rectal GISTs that even received adjuvant Imatinib. The relatively lower malignancy of GISTs in the stomach (1, 32) and higher aggressiveness in the rectum (33, 34) might contribute to these results, which require further investigation focusing on the impact of R1 on the survival of GISTs at different sites. It is clear that the resection margin should not be sacrificed to preserve the organ for at least rectal GISTs. Neo-Imatinib treatment has been reported to reduce the rate of positive margins and is associated with a higher rate of anal preservation for rectal GISTs (35). However, a study by Cavnar_Neo-IM 2020, in which patients all received neo-Imatinib, showed that reduction of tumor size after neo-Imatinib occurred in only 40% of patients and was not associated with better oncologic outcomes. The sensitivity analysis confirmed that omitting this study did not differ from the estimated OS results in the current study. Nevertheless, neo-Imatinib is still recommended for patients with a high potential risk of incomplete resection evaluated preoperatively. Additional attention and treatment are warranted for rectal GISTs when R1 margin occurs.

The current study has some limitations. First, the majority of the included studies were retrospectively designed such that bias was inevitable in the process of this meta-analysis. Second, adjuvant Imatinib was not given specifically to those who experienced R1 margin, so the mechanism of Imatinib attenuating the negative survival impact of R1 needs further exploration. Third, a relatively high heterogeneity was observed in the gastric subgroup without adjuvant Imatinib (P = 0.16, I^2^ = 50%), which makes the result of this subgroup less reliable and requires further exploration. Fourth, risk factors that are collinear with the R1 margin were not analyzed in the current study. In summary, further high-quality case-controlled observational trials with a balanced baseline are needed.

### Conclusions

In comparison with R0 resection, R1 was associated with poor DFS for primary GISTs, which was attenuated by adjuvant therapy with Imatinib. A similar effect of adjuvant Imatinib was observed in the gastric GISTs subgroup. However, rectal GIST patients with R1 resection had poor DFS even when they received adjuvant Imatinib, which suggests that these patients require further investigation. Patients who underwent R1 resection had similar OS compared with those underwent R0 resection regardless of the use of adjuvant Imatinib.

## Data Availability Statement

The original contributions presented in the study are included in the article/**Supplementary Material**. Further inquiries can be directed to the corresponding authors.

## Author Contributions

Concept and design: WK and FF. Literature search and extracting of data: ZL, YZ, HY, XG and JZ. Analyzing and interpretation of data: ZL, SL and ZZ. Drafting of the manuscript: ZL. Critical revision of the manuscript: XY and JY. All authors listed have made a substantial, direct, and intellectual contribution to the work and approved it for publication.

## Funding

This study was supported in part by grants from 1. Wu Jieping Medical Foundation (320. 6750.19020 and 320.6750.2020-08-32); 2. CAMS Innovation Fund for Medical Sciences (2020-I2M-C&T-B-027); 3. Beijing Bethune Charitable Foundation (WCJZL202106); 4. Beijing Xisike Clinical Oncology Research Foundation (Y-HS2019-43).

## Conflict of Interest

The authors declare that the research was conducted in the absence of any commercial or financial relationships that could be construed as a potential conflict of interest.

## Publisher’s Note

All claims expressed in this article are solely those of the authors and do not necessarily represent those of their affiliated organizations, or those of the publisher, the editors and the reviewers. Any product that may be evaluated in this article, or claim that may be made by its manufacturer, is not guaranteed or endorsed by the publisher.

## Supplementary Material

Supplementary Figure 1 Sensitivity analysis of **(A)** disease-free survival and **(B)** overall survival.

## Abbreviations

GIST, gastrointestinal stromal tumor; R1, microscopically positive resection margin; R0, microscopically negative resection margin; DFS, disease-free survival; OS, overall survival; ESMO, European Society for Medical Oncology; NOS, Newcastle–Ottawa Quality Assessment Scale; RCT, Randomized Controlled Trial.
