# Corrigendum: Comparison of prognosis between microscopically positive and negative surgical margins for primary gastrointestinal stromal tumors: A systematic review and meta-analysis

**DOI:** 10.3389/fonc.2022.1110168

**Published:** 2023-01-23

**Authors:** Zhen Liu, Yichunzi Zhang, Han Yin, Xiuzhu Geng, Sishang Li, Jinrong Zhao, Ziyang Zeng, Xin Ye, Jianchun Yu, Fan Feng, Weiming Kang

**Affiliations:** ^1^ Department of General Surgery, Peking Union Medical College Hospital, Chinese Academy of Medical Sciences & Peking Union Medical College, Beijing, China; ^2^ National Health Commission (NHC) Key Laboratory of Systems Biology of Pathogens and Christophe Mérieux Laboratory, Institute of Pathogen Biology, Chinese Academy of Medical Sciences & Peking Union Medical College, Beijing, China; ^3^ Ministry of Health (MOH) Key Laboratory of Systems Biology of Pathogens, Institute of Pathogen Biology, and Center for Tuberculosis Research, Chinese Academy of Medical Sciences & Peking Union Medical College, Beijing, China; ^4^ Department of Hematology, Peking Union Medical College Hospital, Chinese Academy of Medical Sciences & Peking Union Medical College, Beijing, China; ^5^ Division of Digestive Surgery, Xijing Hospital of Digestive Diseases, The Air Force Medical University, Xi’an, China

**Keywords:** gastrointestinal stromal tumor, R1 margin, Imatinib, prognosis, meta-analysis

**Incorrect Affiliation**

In the published article, there was an error in affiliation 1. Instead of “Chinese Academy of Medical Sciences & Peking Union Medical College, Beijing, China,” it should be deleted.

The authors apologize for this error and state that this does not change the scientific conclusions of the article in any way. The original article has been updated.

