# Supplementary figures and images for: Comparison of Prognosis Between Microscopically Positive and Negative Surgical Margins for Primary Gastrointestinal Stromal Tumors: A Systematic Review and Meta-Analysis
Source: Front Oncol. 2022 Apr 19;12:679115. doi: 10.3389/fonc.2022.679115 (PMC9062001; doi:10.3389/fonc.2022.679115)

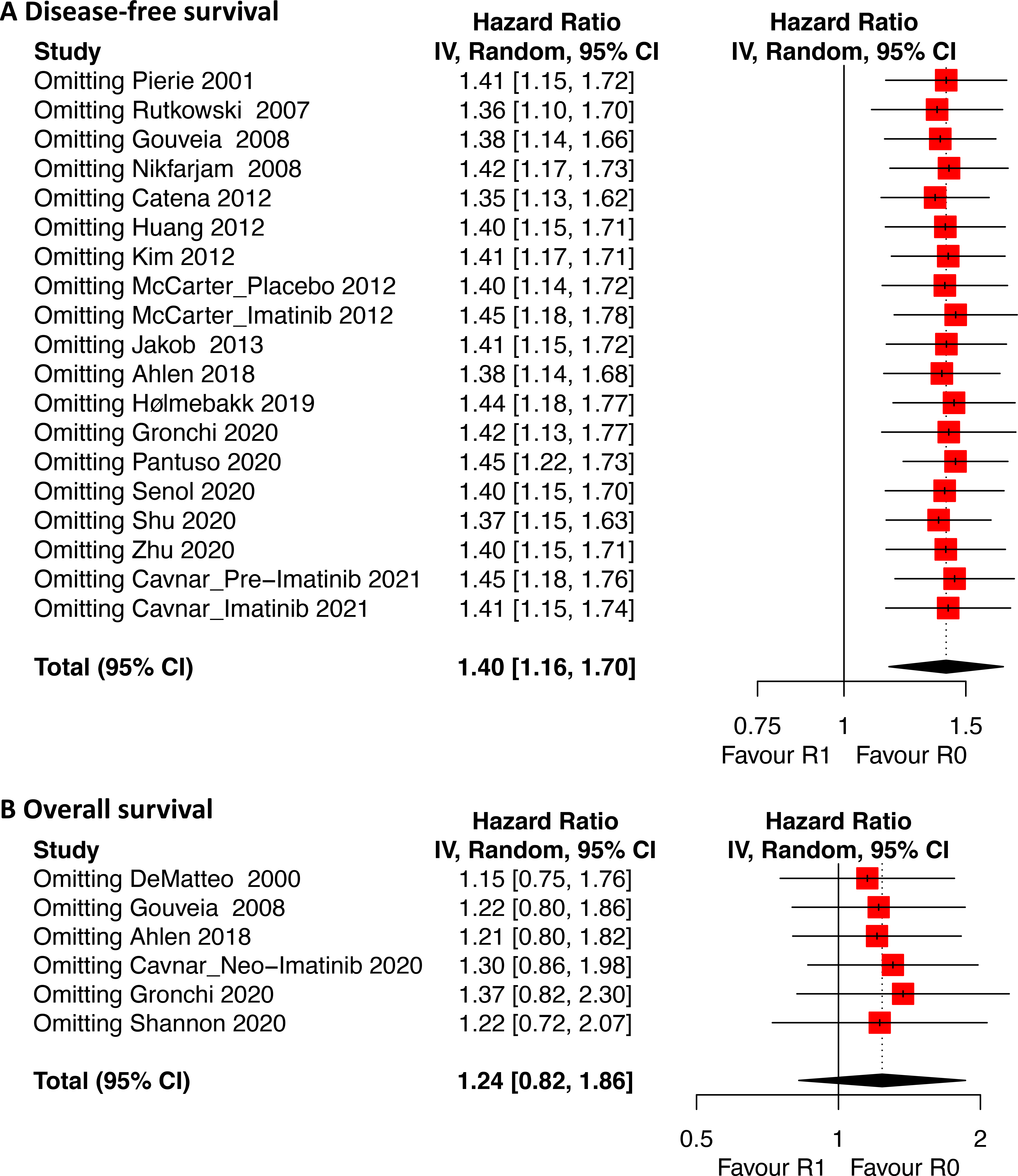

Supplement: Supplementary Figure 1 — Sensitivity analysis of (A) disease-free survival and (B) overall survival. [file Image_1.tif]
